# The Resistance Mechanism Governs Physiological Adaptation of *Escherichia coli* to Growth With Sublethal Concentrations of Carbapenem

**DOI:** 10.3389/fmicb.2021.812544

**Published:** 2022-01-31

**Authors:** Franca Schäfer, Pia Görner, Sabrina Woltemate, Christina Brandenberger, Robert Geffers, Stefan Ziesing, Dirk Schlüter, Marius Vital

**Affiliations:** ^1^Institute for Medical Microbiology and Hospital Epidemiology, Hannover Medical School, Hanover, Germany; ^2^Institute of Functional and Applied Anatomy, Hannover Medical School, Hanover, Germany; ^3^Genomics Research Group, Helmholtz Centre for Infection Research, Braunschweig, Germany

**Keywords:** antibiotic resistance, *Escherichia coli*, ecology, physiology, RNA-Seq

## Abstract

Factors governing resistance in carbapenem-resistant *Enterobacteriaceae* are manifold. Despite ample research efforts, underlying molecular mechanisms are still only partly understood. Furthermore, little is known on (eco)physiological consequences from resistance acquisition originating from distinct mechanisms in respective bacteria.

In this study, we examined physiological adaptation of *Escherichia coli* clinical isolates exhibiting two distinct resistance mechanisms–either carrying a carbapenemase (*n* = 4, CARB) or alterations in porin-encoding genes (*n* = 6, POR)–during growth with sublethal concentrations of ertapenem in chemostat culture. Basic growth parameters based on optical density and flow-cytometric analyses as well as global gene expression patterns using RNA-Seq were recorded. We demonstrate that strategies to deal with the antibiotic were distinct between strains of the two groups, where (increased) expression of carbapenemases was the major response in CARB, whereas wide-spread alterations in gene-expression that promoted a survival-like phenotype was observed in POR. The response in POR was accompanied with “costs of resistance” resulting in reduced growth efficiencies compared with CARB that are intrinsic to that group and were also observed during growth without antibiotic challenge, however, at lower levels. All strains showed similar minimal inhibitory concentrations and did not form phylogenetic groups, indicating that results cannot be attributed to distinct resistance levels or phylogenetic relationships, but are indeed based on the resistance mechanism.

## Introduction

Gram-negative bacteria are a common cause of life-threatening diseases with *Enterobacteriaceae*, such as *Klebsiella* spp. and *Escherichia coli*, playing a major role (Vincent et al., 2009). Immediate effective antimicrobial treatment is highly important, particularly during sepsis, and broad-spectrum antibiotics such as carbapenems are regularly used in first-line therapy (Rhodes et al., 2017). However, an increase in carbapenem-resistant *Enterobacteriaceae* (CRE) has been reported in numerous countries thwarting effective therapy strategies and worsening patients’ outcome (Nordmann and Poirel, 2014).

Carbapenem-resistant *Enterobacteriaceae* show a broad spectrum of resistance mechanisms against carbapenems. Most prominent are carbapenemases that act by cleaving the antibiotic. They belong to three of the four Ambler classes of β-lactams: class A carbapenemases includes KPC, GES, IMI, SME, class B contains the metallo-β-lactamases (VIM, IMP, NDM), and class D represent the OXAs, among which OXA-48 is the most frequent carbapenemase in *Enterobacteriaceae* (Nordmann and Poirel, 2014). Another prominent resistance mechanism to carbapenems are alterations of the main porins OmpC and OmpF (Nikaido, 2003). Porin loss, their reduced expression or changes in electrostatics and channel size, all result in a decreased permeability of the outer membrane for carbapenems, diminishing their effects (Mmatli et al., 2020). In those strains, antibiotic influx is usually not completely blocked, but only reduced (Nikaido, 2003; Ferenci, 2005). A combination of additional mechanisms, such as expression of extended-spectrum β-lactamases (ESBLs) or enzymes of the AmpC-group, increased antibiotic efflux, and target modification of penicillin binding proteins, are believed to contribute to carbapenem resistance in those strains (Adler et al., 2016; Mmatli et al., 2020).

A better understanding on CRE’s resistance mechanisms and their (eco)physiologic consequences will offer chances to develop new and precise intervention strategies to restrict the spread of CREs and reduce infections with those harmful bacteria. Several studies have investigated the consequences of antibiotic resistance for bacterial fitness. By reviewing the literature Vogwill and Mclean suggest on average lower fitness costs of plasmid-encoded resistance, including most carbapenemases, over chromosomally encoded mechanisms, such as porin alterations (Vogwill and MacLean, 2015). However, the authors showed that cost of resistance is highly variable between strains and specific conclusion for individual mechanistic groups remained largely elusive. Mathieu et al. further demonstrated a general core hormetic stress response in reaction to sublethal antibiotic stress for several β-lactams (Mathieu et al., 2016). Others reported that gene expression responses vary depending on the expressed carbapenemase (Sidjabat et al., 2018), and alterations of main porins were reported to be accompanied by a reduction in maximum specific growth rates (Knopp and Andersson, 2015). Overall, detailed investigations on the impact of the resistance mechanisms against carbapenems on bacterial (eco)physiology are not available.

In this study, we used clinical isolates of *E. coli* comparing carbapenemase (OXA-48) carrying strains with those exhibiting porin-alterations, i.e., non-functional OmpC/F encoding genes. Physiological adaptation strategies between the groups during growth with sublethal concentrations of ertapenem in chemostat cultures were investigated applying flow cytometric-based techniques and global transcriptome analyses (RNA-Seq). All strains exhibited similar minimal inhibitory concentrations (MICs) and were phylogenetically diverse (no grouping with the resistance-mechanism) in order to exclude any phylogenetically based confounders.

## Materials and Methods

### Bacterial Strains and Culture Conditions

We sequenced in total 50 carbapenem resistant *E. coli* strains isolated from patients between 2013 and 2019 during routine diagnostics of the Institute of Medical Microbiology and Hospital Epidemiology of Hannover Medical School, Hannover, Germany. Ten strains exhibiting distinct resistance mechanisms that are either based on presence of a carbapenemase gene (*oxa-48*) (*n* = 4) or alterations (partial deletions) in the porin-encoding genes *ompC/F* (*n* = 6) were included in this study. Strains were specifically selected to avoid phylogenetic coherence within resistance groups and assure a narrow MIC range from 2 to 4 mg L^–1^ (strain EC1 had a MIC of 1 mg L^–1^) for ertapenem. All Experiments were approved by the local ethics committee (#9399_BO_K_2020).

The experimental set-up for continuous culturing under carbon-limited conditions was performed as previously described (Vital et al., 2014) using a minimal medium (Ihssen and Egli, 2004) containing 1 g L^–1^ glucose and 0.5 g L^–1^ casamino acids. For experiments including ertapenem (INVANZ, MSD, Germany), the antibiotic was added to the feed medium at a concentration of 0.5 mg L^–1^. Cryo-cultures of strains were streaked onto LB agar plates (BD Difco LB Agar, Merck, Germany) containing 0.5 mg L^–1^ ertapenem and were subsequently grown in minimal medium (+/− ertapenem) overnight. One mL of this culture was inoculated into reactors and grown in batch-mode over night before continuous culturing (switch-on of the pump) was started at a dilution rate of 0.25 h^–1^. Samples were harvested at steady state growth, defined as constant biomass [measured by optical density (OD)] over time, which was achieved after 5–10 volume changes. Single cultures were analyzed for each strain (+/− ertapenem) and all strains of a group were treated as replicate samples in follow-up analyses.

### Sample Preparation/Measurements, Minimal Inhibitory Concentration (MIC) Determination, and Transmission Electron Microscopy (TEM) Analysis

Biomass was recorded by *OD*_600_ measurements (Genesys 20, Thermo Fisher Scientific, United States), while cell concentrations were determined by flow cytometry (MACSQuant Analyzer, Miltenyi Biotec, Germany) using SYBR Green I staining (Thermo Fisher Scientific, United States) (Hammes et al., 2008). Relative cell size and relative DNA content were based on signal extents of the sideward-scattering light and of green fluorescence, respectively. For transcriptomics, 500 μl of cultures were directly added to RNAlater (Thermo Fisher Scientific, United States) and stored at −80°C until RNA extraction. For residual substrate measurements 1 mL of cultures were spun down for 3 min at 13.000 rpm (4°C) and the supernatant was subjected to glucose (Glucose-Glo Assay, Promega, United States) and amino-acid (L-Amino Acid Quantitation Kit, Merck, Germany) determination according to the manufacturers’ protocols. The pH was measured as a control parameter and cultures were regularly monitored for possible contamination by plating dilutions on LB agar plates followed by taxonomic classification of colonies based on MALDI-TOF (AXIMA Assurance, Shimadzu, Japan). For this purpose, a bacterial colony was spotted onto a polished steel MALDI-TOF target plate (BioMérieux, Nuertingen, Germany) and 1 μl of matrix (CHCA, alpha-cyano-4-hydroxy-cinnamic acid) was added. The Saramis database (v4.16) was used for classification.

Minimal inhibitory concentration (MIC) were determined from overnight cultures without any antibiotics, which were diluted with saline to 0.5 McFarland and subjected to VITEK 2 AST cartridges (Biomérieux, France). For assessing MIC values specifically for our culture conditions, we applied the micro dilution method, where 96 deep well plates were loaded with 500 μl minimal medium containing different concentrations of ertapenem (0, 0.5, 1, 2, 4, 6, 8, and 10 mg L^–1^). Overnight cultures without any antibiotic were diluted with saline to 0.5 McFarland and used as an inoculum (5 μL) in replicate samples; plates were subsequently incubated at 37°C (200 rpm). MIC values were defined as the lowest antibiotic concentration preventing the bacterial growth from reaching an *OD*_600_ higher than 0.12 Â after 48 h (SYNERGY HTX Multi-Mode Reader, BioTek, United States) and additional visual inspections of turbidity were performed.

For transmission electron microscopy (TEM) analyses bacterial cells (2 mL) were pelleted by centrifugation and fixated with a mixture of 1.5% paraformaldehyde (Merck, Germany) and 1.5% glutaraldehyde (Merck, Germany) in 0.15 M HEPES buffer (Merck, Germany) for 24 h. Embedding and slicing was performed as described previously (Kling et al., 2017). Sections were then investigated with a Morgagni 268 microscope (FEI, United States).

### Genome Sequencing and Bioinformatics Analyses

DNA was extracted (DNeasy PowerSoil Pro Kit, Qiagen, Germany) and libraries were prepared (Illumina DNA Prep, Illumina, United States) and subsequently sequenced on Illumina NovaSeq 6000 (at Helmholtz Centre for Infection Research (HZI) in paired-end mode (2 × 150 bp) to achieve a coverage >50×. For Oxford Nanopore (ONP) sequencing, DNA was extracted using the Genomics-tip 100/G kit (Qiagen, Germany), subjected to the rapid barcoding kit (ONP, United Kingdom), and subsequently sequenced on a MinION flow cell using a MinION device. Basecalling of recorded FAST5 files was done using Guppy in MinKNOW (v20.06.4).

Hybrid assemblies were constructed from reads of both sequencing techniques using Unicycler (v0.4.8; *default mode*) (Wick et al., 2017); ONP reads > 1 kb were included into analyses. Assembled genomes were then uploaded to the RAST server that provides gene calling and annotation based on SEED’s Subsystems Technology (Aziz et al., 2008). Average nucleotide identities (ANI) were calculated using the fastANI tool (v1.32; *default mode*) (Jain et al., 2018) and number of shared genes was determined based on the program roary (v3.13.0; *default mode*) (Page et al., 2015). Raw Illumina reads were subjected to ARIBA (v2.14.6; *default mode*) (Hunt et al., 2017) using the CARD database (version July 2021) (Alcock et al., 2020) for detection of antibiotic resistance genes (β-lactamases) and completeness of genes encoding porins. *OmpC* and *ompF* genes from the RAST output were additionally manually inspected for specific deletion sites in JalView (v. 2.11.1.3) (Waterhouse et al., 2009).

### RNA-Seq and Follow-Up Analyses

RNA was extracted (RNeasy Mini Kit, Qiagen, Germany) followed by DNase I treatment (RNase-Free DNase Set, Qiagen, Germany) and quality controlled (RIN value) using a 4200 TapeStation (Agilent Technologies, United States). Ribosomal RNAs were depleted by the NEBNext Bacterial rRNA Depletion Kit (NEB, United States) before generating libraries using the NEBNext Ultra II Directional RNA Library Prep Kit (NEB, United States) with NEBNext Multiplex Oligos for Illumina (NEB, United States). Libraries were purified using AMPure beads (Beckman Coulter, United States) according to the NEB protocol and sequenced on an Illumina NovaSeq 6000 (at HZI) in paired-end mode (2 × 150 bp; ∼5 × 10^6^ per sample).

Raw reads were quality filtered and depleted of sequences derived from rRNA (using a custom database based on our genomes) using KneadData (Huttenhower lab; v0.7.2) and subsequently mapped to genomes using BBMap (from JGI; v38.22; paired-end mode). Transcript coverage files were calculated (*pileup.sh* script from BBMap) and loaded into R (v. 4.1.0) to calculate Transcripts Per Million (TPM) and fold changes between conditions for all strains. Ordination (Principal Component Analysis) and hierarchical cluster analysis (complete linkage clustering based on Euclidean distances) were done on square-root transformed TPM data of shared genes using *vegan* (v2.5.7). Growth parameters (OD, cell concentration, relative cell size and DNA content) and expression data (barplots) were visualized using *ggplot2* (v3.3.5). Student’s *t*-test was performed in R (*t.test*) on log10 transformed data, whereas differential expression analyses of genes and on all SEED subsystem levels (cumulative abundances) were done with DESeq2 (v1.32.0) (Love et al., 2014) on raw count data treating all strains of a group as replicates. A fold change of 1 was defined as the ratio of 2 in gene expression data (TPM) between two conditions (higher value/lower value).

All bioinformatics analyses were performed on MHH’s High-Performance Computer Sequencing Cluster (HPCSeq).

## Results

We performed experiments in chemostat culture, i.e., steady-state growth in continuous culture, with and without the addition of ertapenem at sublethal concentrations in order to elucidate physiological adaptations of *E. coli* to grow with carbapenems. Throughout this study we were specifically interested in response differences between bacteria exhibiting a carbapenemase gene (*oxa-48*) (*n* = 4) and those characterized by alterations (partial deletions) in the porin-encoding genes *ompC/F* (*n* = 6), referred to as CARB and POR, respectively, in the following text.

**FIGURE 1:**
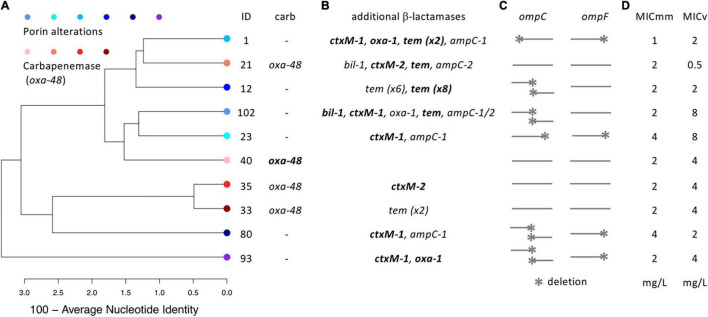
A dendrogram based on average nucleotide identities (ANI) of *E. coli* isolates of the two resistance groups [carbapenemase (*n* = 4) vs porin alterations (*n* = 6)] are shown in panel **(A)** (carb: carbapenemase). Detected genes encoding additional β-lactamases are indicated in panel **(B)**, where plasmid encoded genes are highlighted in bold (genes detected multiple times are indicated). Deletion sites in *ompC/F* genes are shown in panel **(C)**. A graphical outline of respective assembles are given in Supplementary Figure 1. Minimal inhibitory concentrations (MIC) values for ertapenem are given in panel **(D)** (“MICv” is based on standard VITEK 2 results, whereas “MICmm” was determined using the minimal medium of this study).

We included strains displaying no phylogenetic coherence within resistance groups and exhibiting a narrow MIC range from 2 to 4 mg L^–1^ (strain EC1 had a MIC of 1 mg L^–1^) for ertapenem (Figure 1). In the POR group, three strains had a deletion only in the *ompC* gene while the other three had alterations in both *ompC* and *ompF* genes. All CARB strains showed intact *ompC* and *ompF* genes. Applying hybrid assembly on Illumina short reads and Oxford Nanopore-derived long-reads, we could close nine of the ten genomes (EC102 displayed a large contig of 4.8 Mb) and demonstrated the presence of plasmids in all strains (Supplementary Figure 1). Only one OXA-48 was plasmid encoded and a heterogenic distribution of β-lactamases, that were largely present on plasmids, were detected with no clear differences between the two groups; strains exhibited 0 to 6 different types of β-lactamases (Figure 1). The following β-lactamase encoding genes were detected in CARB: EC21 [*oxa-48, ctx-M-1, oxa-1, tem* (x2), *ampC-1*], EC33 [*oxa-48, tem* (x2)], EC35 (*oxa-48, ctxM-2*), EC40 (*oxa-48)*, and POR: EC01 [*ctx-M-1, oxa-1, tem* (x2), *ampC-1*], EC12 [*tem* (x14)], EC23 (*ctx-M-1, ampC-1*), EC80 (*ctx-M-1, ampC-1*), EC93 (*ctx-M-1, oxa-1*), EC102 (*bil-1, ctx-M-1, oxa-1, tem, ampC-1/2*), strains.

### Basic Physiological Parameters–POR Formed Fewer, but Bigger Cells With Increased DNA Content

Pre-experiments indicated that the maximum specific growth rate (μ_max_) differed between strains and was affected by antibiotic challenge (Supplementary Figure 2) demonstrating the necessity of continuous culture growth that maintains the same growth rate across strains and conditions (+/− ertapenem) in order to exclude confounding results from this parameter.

**FIGURE 2:**
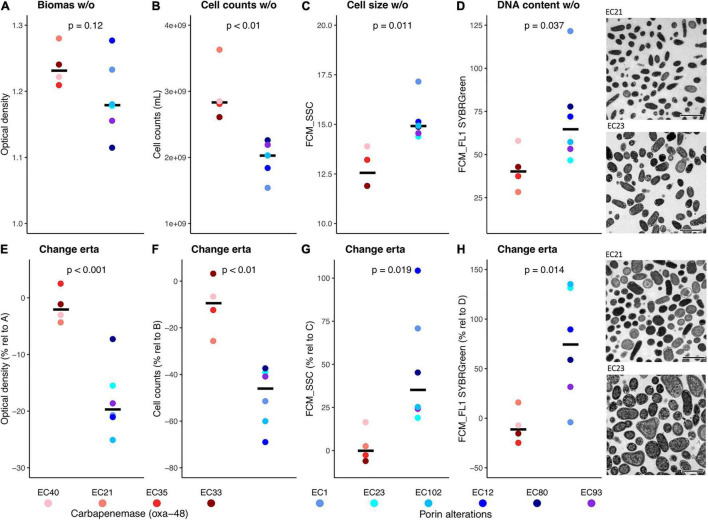
Results of basic growth parameters of strains of the two groups determined during steady-state growth in continuous culture are shown. The upper row (panels **A–D**) displays results during growth without carbapenem (w/o). **(A)** biomass (OD measurements), **(B)** cell concentrations [based on flow cytometric measurements (FCM)], **(C)** relative cell size (based on FCM sideward scatter signals; values of EC21 and EC33 are very similar and undistinguishable on the plot), **(D)** relative DNA content (based on FCM green fluorescence signals). The lower row (panels **E–H**) displays results during growth with sublethal concentrations of carbapenem (0.5 mg L^–1^ ertapenem; erta) relative to growth without the antibiotic. Results of statistical testing (Student’s *t*-test) between groups are indicated. Transmission electron microscopy pictures of representative strains of each group during growth in both conditions (+/− ertapenem) are shown on the right. The scale bar represents 2 μm.

Steady-state growth was monitored by OD measurements accompanied by flow cytometric analyses yielding cell concentrations and estimations on relative cell size as well as relative DNA content. As shown in Figure 2, those parameters already differed between the two groups without any antibiotic stress, where the POR strains displayed reduced concentrations of cells that showed increased relative cell sizes and higher relative DNA contents; OD was not significantly different. Thus, the resistance mechanism affected growth even without the addition of any antibiotics. Confronted with sublethal concentrations of ertapenem (0.5 mg L^–1^), only the POR strains showed significant changes in growth parameters displaying lower biomass (OD) (−18.04 ± 6.14%) and fewer cell concentrations (−49.61 ± 12.84%) with increased relative cell sizes (+48.13 ± 33.56%) and higher relative DNA contents (+73.78 ± 55.51%) (Figure 2). TEM of cultures from two selected strains [EC21 (CARB) and EC23 (POR)] confirmed flow cytometry-based results on cell size differences (Figure 2). CARB strains were unaffected by the addition of the antibiotic. As a result, differences between the two groups were increased for all parameters during antibiotic pressure, where POR strains displayed on average an OD of 79.83%, cell numbers of 66.61%, a relative cell size of 172.64%, and a relative DNA content of 304.60% compared with CARB strains (data not shown). We did not detect any residual glucose or amino acid concentrations in supernatants of cultures demonstrating C-limited growth under both growth conditions.

### Transcriptomics–Higher Responses to Ertapenem in the POR Group

**FIGURE 3:**
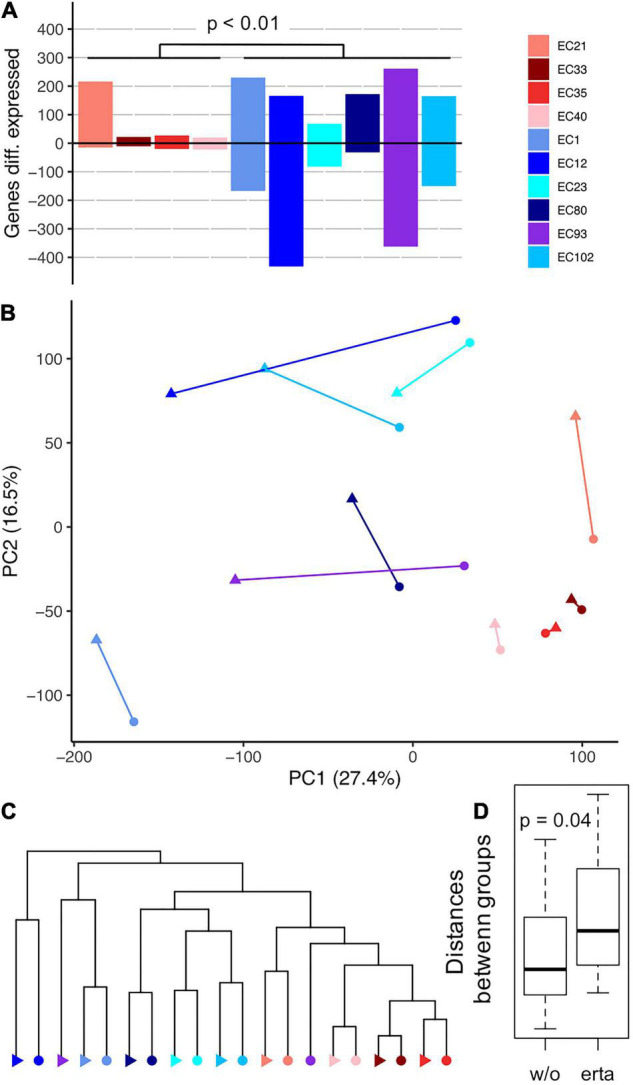
Gene expression results of strains grown in continuous culture in steady-state (+/− ertapenem) are shown. Panel **(A)** displays amount of differentially expressed genes of individual strains during growth with the antibiotic compared with growth without ertapenem. All genes that showed a TPM >10 and a fold change >1 were considered; the *p*-value (Student’s *t*-test) refers to amount of differentially expressed genes between the two groups. A principal component analysis based on expression patterns of shared genes is shown in panel **(B)** and results of hierarchical clustering are shown in panel **(C)** (circles and triangles represent results from growth with and without ertapenem, respectively). Euclidean distances of all pair-wise comparisons between strains of individual groups (−/+ ertapenem; w/o/erta) is shown in panel **(D)** (*p*-value was based on Student’s *t*-test result).

For gaining deeper insights into cell’s physiology, we examined the transcriptome of every strain grown with and without ertapenem. All strains shared 3195 genes, referred to as “the shared genome,” which represents on average 61.25 ± 3.11% of a strain’s genome (Supplementary Figures 3A,B). As expected, the shared genome was much higher expressed (+722%) than other genes belonging to the so-called “accessory genome” (Supplementary Figure 3C). Overall, strains of the POR group displayed significantly more differentially expressed genes, defined as genes with a TPM >10 and a fold change >1 (+/− ertapenem), namely, 381 ± 198, than CARB strains (88 ± 95) (Figure 3A). Of those genes 57.84 ± 6.29% (POR) and 41.81 ± 18.05% (CARB) belonged to the shared genome.

Principal component analysis (PCA) and hierarchical clustering based on gene expression profiles of shared genes indicated a strong clustering for each strain and a clear division into the resistance groups, where all strains of the CARB group clustered together, even without ertapenem (Figures 3B,C). An exception was EC93 (without ertapenem) that interleaved CARB strains in the dendrogram. Importantly, this coherent clustering of strains, based on their resistance mechanism, did not reflect their phylogenetic relatedness (compare Figure 1). The observed higher transcriptional responses of POR strains compared with CARB strains to ertapenem exposure (Figure 3) was in accordance with growth parameters shown in Figure 2. Based on PCA analysis, all POR strains altered their gene expression in a similar manner diverging further from the CARB group, as the latter showed only little changes in gene expression, except for EC21. Accordingly, the average Euclidean distance between the two groups was increased during growth with the antibiotic (Figure 3D).

### Expression of Genes Expected to Play a Key Role in Carbapenem Resistance

**FIGURE 4:**
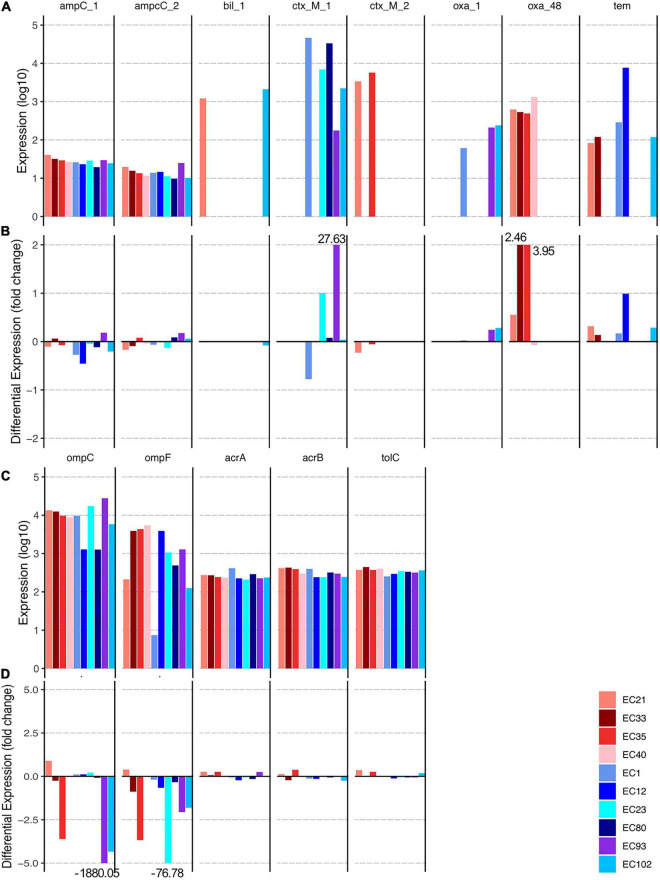
(Differential) expression results of genes reported to play a key role in resistance to carbapenems are shown. Panels **(A,B)** give expression results of genes encoding ß-lactamases, including the carbapenemase Oxa-48, whereas expression of genes encoding the porins OmpC and OmpF and of proteins that form the AcrAB-TolC efflux pump are shown in panels **(C,D)**.

We explored expression of genes that encode enzymes that are known to be involved in carbapenem-resistance (Figure 4). In particular, we focused on all detected genes encoding β-lactamases (*ampC-1/2*, *bil*, *ctx-M-1/2*, *oxa-1*, *tem*), including the carbapenemase *oxa-48*, and those encoding porins (*ompC*, *ompF*) as well as on *arcA/B, tolC* genes that encode the respective efflux pump. While genes of the *ampC* group were lowly expressed in all strains in both conditions, a few β-lactamases considered as ESBLs, as well as all *oxa-48*, were highly expressed during growth, even without ertapenem (Figure 4A). *Ctx-M-1* genes and *tem* of the POR strains EC23, EC93, and EC12, respectively, were increasingly expressed during growth with ertapenem (Figure 4B). Expression of *oxa-48* genes was induced by the antibiotic in three CARB strains, whereas the only plasmid encoded *oxa-48* (from EC21) did not change its expression level. Genes associated with the efflux pump were unaffected, while both porin-encoding genes of EC33 (CARB group) and of EC1, EC23 (only *ompF*), EC93 (all POR group) were down-regulated during growth with ertapenem (Figures 4C,D).

### Specific Physiological Adaptation Strategy of the POR Group Strains During Growth With Ertapenem

**FIGURE 5:**
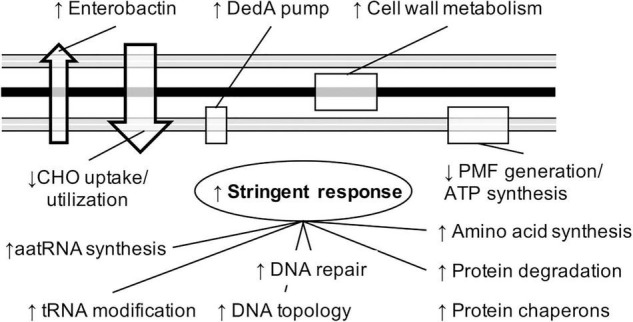
A schematic model describing physiological adaptation strategies of strains exhibiting porin alterations during growth with sublethal concentrations of ertapenem based on results of this study is shown. Arrows indicate up-/down-regulation of genes encoding respective cellular processes during growth with the carbapenem. For explanations see text. PMF, proton motive force; CHO, carbohydrates.

Global differential expression analyses based on DESeq2 did reveal only three genes significantly affected by the carbapenem in the CARB group, while a multitude of significantly differentially expressed genes (*n* = 462) and various subsystem levels were detected for the POR group (Supplementary Table 1). Those findings are in accordance with results shown in Figure 3A. We took a closer look into gene expression responses to growth with ertapenem in the POR group and constructed a mechanistic model based on our results (Figure 5).

We detected an overall increased expression of the Subsystem “*Stringent Response*” (*p.adj* = 0.023) with associated genes, namely, the two GTP pyrophosphokinases (*relA*) and Guanosine-3′, 5′-bis(diphosphate) 3′-pyrophosphohydrolase (*spoT*), increasingly expressed in all strains (expression of individual genes did, however, not reach statistical significance; *p.adj* > 0.05) (Supplementary Table 1). Guanosine-5′-triphosphate, 3′-diphosphate pyrophosphatase (*gppA*) was only increased in four of the strains. Accordingly, a shift in major cellular processes that can be linked to an up-regulation of the stringent response was observed, such as the Subcategories “*DNA repair*” (*p.adj* = 0.049), with the Subsystems “*bacterial RecBCD pathway*” (*p.adj* < 0.01), “*bacterial MutL-MutS system”* (*p.adj* < 0.01) and “*DinG and relatives”* (*p.adj* < 0.01), and processes involved in DNA topology, including the Subsystem “*DNA topoisomerases, Type I, ATP-independent”* (*p.adj* = 0.039) and the Role “*DNA gyrase subunit B (EC 5.99.1.3)*” (*p.adj* = 0.047). Furthermore, an up-regulation of aminoacyl-tRNA synthetases, namely, *“Phenylalanyl-tRNA synthetase alpha and beta chains (EC 6.1.1.20)”* (both *p.adj* < 0.01), *“Histidyl-tRNA synthetase (EC 6.1.1.21)”* (*p.adj* = 0.031), *“Isoleucyl-tRNA synthetase (EC 6.1.1.5)*” (*p.adj* = 0.053) as well as of the tRNA modifying enzymes *“tRNA [guanosine(18)-2′-O]-methyltransferase (EC 2.1.1.34)”* (*p.adj* = 0.044) and “*tRNA dimethylallyltransferase (EC 2.5.1.75)”* (*p.adj* < 0.01), was observed. Additionally, the Subcategory “*Proteolysis in bacteria, ATP-dependent*” (*p.adj* = 0.025), along with several Subsystems involved in amino acid synthesis [e.g., *“Lysine Biosynthesis DAP Pathway”* (*p.adj* = 0.038), “*Tryptophan synthesis*” (*p.adj* < 0.01), “*Glutamine synthetases*” (*p.adj* < 0.01)], and the subsystem “*Protein chaperones*” (*p.adj* < 0.01) were up-regulated (Supplementary Table 1).

Our results further indicate major rearrangements in the periplasmatic space as genes of the Category “*Cell Wall and Capsule”* (*p.adj* = 0.015) involving the Subsystem “*Murein Hydrolases*” (*p.adj* = 0.044) and the Roles *“UDP-N-acetylglucosamine 1-carboxyvinyltransferase (EC 2.5.1.7)*” (*p.adj* = 0.019), “*Glucosamine-1-phosphate N-acetyltransferase (EC 2.3.1.157)”* (*p.adj* = 0.011) and “*Periplasmic thiol:disulfide interchange protein DsbA*” (*p.adj* < 0.01), were increasingly expressed. This went along with an overall down-regulation of several genes encoding enzymes involved in building a proton-motive force and the generation of ATP *via* aerobic respiration, namely, *“Cytochrome O ubiquinol oxidase subunits I, II, and III (EC 1.10.3.-)”* (*p.adj* = < 0.01), *“Cytochrome d ubiquinol oxidase subunit I and II (EC 1.10.3.-)”* (*p.adj* < 0.01) together with “*Formate dehydrogenase O alpha, beta and gamma subunits (EC 1.2.1.2)”* (*p.adj* < 0.01–0.041), *“D-lactate dehydrogenase (EC 1.1.1.28)”* (*p.adj* = 0.031), “*Glycerol-3-phosphate dehydrogenase [NAD(P)+] (EC 1.1.1.94)”* (*p.adj* = 0.065), and “*ATP synthase F0 sector subunit a (EC 3.6.3.14)”* (*p.adj* = 0.024).

Furthermore, higher transcript levels of the Subsystem “*Siderophore Enterobactin*” (*p.adj* = 0.013) and the Role “*DedA family inner membrane protein YqjA*” (*p.adj* < 0.01) were detected, whereas Subsystems involved in uptake and catabolism of several carbohydrates, namely, “*L-Arabinose utilization*” (*p.adj* < 0.01), “*L-fucose utilization*” (*p.adj* = 0.014), “*Maltose and Maltodextrin Utilization*” (*p.adj* = 0.017), “*D-gluconate and ketogluconates metabolism*” (*p.adj* = 0.018), “*D-galactonate catabolism*” (*p.adj* = 0.025), “*Glycerol and Glycerol-3-phosphate Uptake and Utilization*” (*p.adj* < 0.01), and “*Lactate utilization*” (*p.adj* = 0.018), were down-regulated.

## Discussion

The global spread of carbapenem-resistant bacteria is increasing and threatens the therapeutic success in infections with *Enterobacteriaceae* (David et al., 2019). Understanding the molecular processes involved in resistance and their consequences on (eco)physiology of those bacteria are a crucial part to develop strategies for limiting spread of resistance and to prevent infection. In this study, we focused on elucidating differences in physiological adaptation mechanisms of clinical isolates of *E. coli* to growth with sublethal concentrations of ertapenem, whose primary resistance mechanisms were either the presence of a carbapenemase (CARB) or alterations in porin encoding genes (POR).

In order to ensure defined growth conditions for all strains in both conditions, i.e., with and without ertapenem, we performed experiments in chemostat culture fed by a minimal medium. This allowed to provide identical growth rates in all experiments, a parameter that is known to affect physiology of *E. coli*, including its stress response (Ihssen and Egli, 2004). Results of batch cultures showed that growth rates indeed varied considerably between strains and differences +/− ertapenem were obvious (Supplementary Figure 2). Furthermore, complex media (e.g., LB medium) provide unstable growth conditions, where easily degradable nutrients are consumed before more complex substrates (Berney et al., 2006). Exact comparisons between strains/conditions, e.g., based on RNA-Seq analyses, can be difficult to achieve in such set-ups as, next to the factor under study (e.g., antibiotic challenge), expression patterns are additionally governed by nutritional conditions present at a given time-point potentially biasing results.

Our experiments revealed substantially different responses to sublethal concentrations of ertapenem in the two resistance groups POR and CARB, which was apparent from growth parameters and gene expression data, where only the POR group displayed strong reactions to antibiotic challenges. Results support the view that carbapenem-resistance in strains that are characterized by porin alterations is not a mere result of reduced influx of the antibiotic (Mmatli et al., 2020). Rather, those structural alterations diminish antibiotic concentrations at target sites and are accompanied by an additional response level that includes wide-ranging cellular mechanisms promoting survival and repair. This is further supported by the fact that deletion of porins alone does not introduce resistance to carbapenems in *E. coli* (Choi and Lee, 2019). While all POR strains carried several ESBL genes, their expression was constitutive with only a few increasing their transcript levels during antibiotic challenge. Their role during antibiotic challenge remained, hence, largely elusive in this study and more research is required on this topic.

Overall, we show that the importance of a global response depends on the nature of the primary resistance level, where presence and (increased) expression of carbapenemases were very effective against antibiotic challenge, without any requirements for additional responses. Whether CARB strains initiate similar global responses as POR when facing higher ertapenem concentrations more closely to the MIC was not investigated (Chetri et al., 2019). Overall, carbapenemase-carrying strains are usually much more resistant to carbapenems compared with those exhibiting other resistance mechanisms (David et al., 2019) and it was, hence, important to choose strains that were characterized by similar MICs and phylogenetically incoherent in order to exclude any confounding effects based on the resistance level or phylogeny. With MIC values around 2–4 mg L^–1^, resistance levels of our strains are considered low and it needs to be seen how results relate to strains that are more resistant to carbapenems.

A closer look into responses of the POR group strains during antibiotic stress revealed several response mechanisms involved (Figure 5). A central role was attributed to the stringent response, a stress response mediated by guanosine pentaphosphate (pppGpp) and guanosine tetraphosphate (ppGpp) (Cashel and Gallant, 1969). The so-called alarmones bind on various proteins and directly modulate their activity. Most wide-ranging effects are achieved by targeting the RNA polymerase (RNAP) resulting in global gene expression changes (Irving et al., 2021). Sanchez-Vazquez et al. investigated RNAP-mediated effects in detail and demonstrated that expression of hundreds of genes were altered that are involved in multiple cellular processes triggering a survival-like phenotype (Sanchez-Vazquez et al., 2019). Many of those processes, such as an induction of genes encoding enzymes involved in DNA damage repair and DNA topology, as well as an up-regulation of genes involved in synthesis of amino acids and aminoacyl-tRNA, along RNA-modifying enzymes, were also recorded here. An observed reduced expression of genes involved in carbohydrate uptake/metabolization and aerobic respiration, along with an increase in transcript levels of genes encoding a potent iron-scavenging chelator, fitted well in our model that describes a survival-like phenotype during growth with ertapenem (Vital et al., 2014; Peralta et al., 2016). Our study additionally indicates that bacterial responses specific in the periplasm were important to POR strains to cope with the antibiotic. Several genes encoding enzymes linked to peptidoglycan metabolism were increasingly expressed, which might be a direct effect of antibiotic action, and an up-regulation of the DedA pump encoded by *yqjA*, that is deemed essential for maintaining envelop homeostasis (Sikdar et al., 2013), was observed.

Strains of the POR group displayed a reduction in biomass (−18.04%) and cell numbers (−49.61%) during steady-state growth with ertapenem that we consider as costs of their resistance strategy, i.e., energy/carbon devoted to a survival-like phenotype. Obvious were also changes in cell morphology, where those strains displayed much bigger relative cell sizes and contained more DNA during growth with the antibiotic. The exact role of ertapenem, which binds to penicillin binding protein 2 (PBP2) that promotes cell elongation, and less on PBP3 that plays a central part in cell division, in this process is not known (Zhanel et al., 2005; Sauvage et al., 2008). Interestingly, cell numbers and morphology in the POR group were significantly distinct from CARB strains already during growth without ertapenem, and both biomass (*p* = 0.12) and maximum specific growth rates (*p* = 0.052) trended to be lower in the POR group. Those observations are in line with the self-preservation and nutritional competence (SPANC) balance theory, where survival mechanisms, including stress resistance, are in a trade-off with efficient nutrient uptake and reproduction (Ferenci, 2005). Our results suggest that intrinsic resistance mechanisms of POR strains directed SPANC toward survival, whereas CARB strains had a more balanced SPANC and were more competent in substrate consumption and growth. As a consequence, global transcription profiles clustered strains into the two resistance groups, even without an antibiotic challenge, demonstrating that global gene expression is largely governed by the resistance mechanism and uncoupled from phylogeny in those strains. While our data suggest that *cis*-regulatory changes in the POR group reduced growth efficiencies, it remains unclear how this relates to the overall fitness. Compensatory mechanisms, specifically, the upregulation of the alternative porins PhoE and ChiP, that have been found in *in vitro* evolution experiments (Knopp and Andersson, 2015), were not detected here (data not shown). However, given that porin loss is a very common feature in patients’ isolates (Nordmann and Poirel, 2019), a reduction in growth yield seems to be an acceptable trade-off, at least in clinical settings, where constant antibiotic challenges, even at low concentrations, select for antibiotic resistant strains (Gullberg et al., 2011). Furthermore, for *Klebsiella pneumoniae* it was recently demonstrated that porin loss promotes increased survival within macrophages, indicating certain fitness advantages for porin-mutant strains in the host (Brunson et al., 2019), even without antibiotic pressure.

In summary, our experiments revealed a strong impact of the carbapenem-resistance mechanism on responses to sublethal concentrations of antibiotics and on bacterial physiology in general. The study should stimulate research on (eco)physiological consequences of resistance acquisition focusing on the underlying resistance mechanism, which can assist risk assessment and the development of precision measures in order to restrict the spread of antibiotic resistance.

## Data Availability Statement

The datasets presented in this study can be found at the European Nucleotide Archive (PRJEB47826).

## Author Contributions

MV and FS designed the study, performed bioinformatics and data analyses, and wrote the manuscript. FS, PG, SW, CB, RG, and MV performed the experiments. All authors commented on the text, contributed to the article, and approved the submitted version.

## Conflict of Interest

The authors declare that the research was conducted in the absence of any commercial or financial relationships that could be construed as a potential conflict of interest.

## Publisher’s Note

All claims expressed in this article are solely those of the authors and do not necessarily represent those of their affiliated organizations, or those of the publisher, the editors and the reviewers. Any product that may be evaluated in this article, or claim that may be made by its manufacturer, is not guaranteed or endorsed by the publisher.
