# Supplementary material for: The Resistance Mechanism Governs Physiological Adaptation of Escherichia coli to Growth With Sublethal Concentrations of Carbapenem
Source: Front Microbiol. 2022 Jan 31;12:812544. doi: 10.3389/fmicb.2021.812544 (PMC8841762; doi:10.3389/fmicb.2021.812544)
Supplement: Supplementary file 2 [file Data_Sheet_1.PDF]

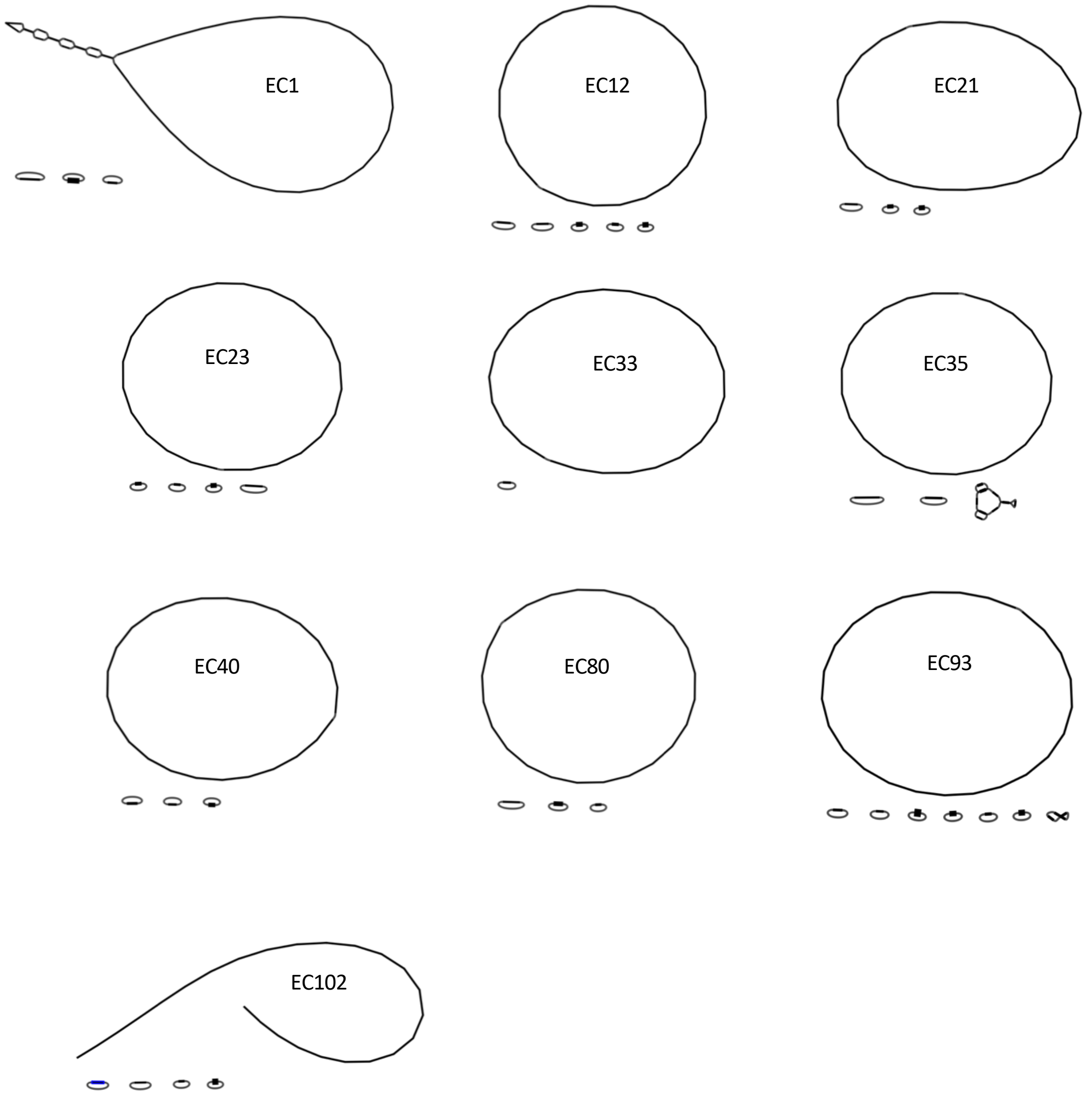

Figure S1. Graphical representation of hybrid assemblies (based on Oxford Nanopore and Illumina outputs) of strains used in this study. The chromosome (large circle) and individual plasmids are indicated; the chromosome of EC102 was not fully closed.

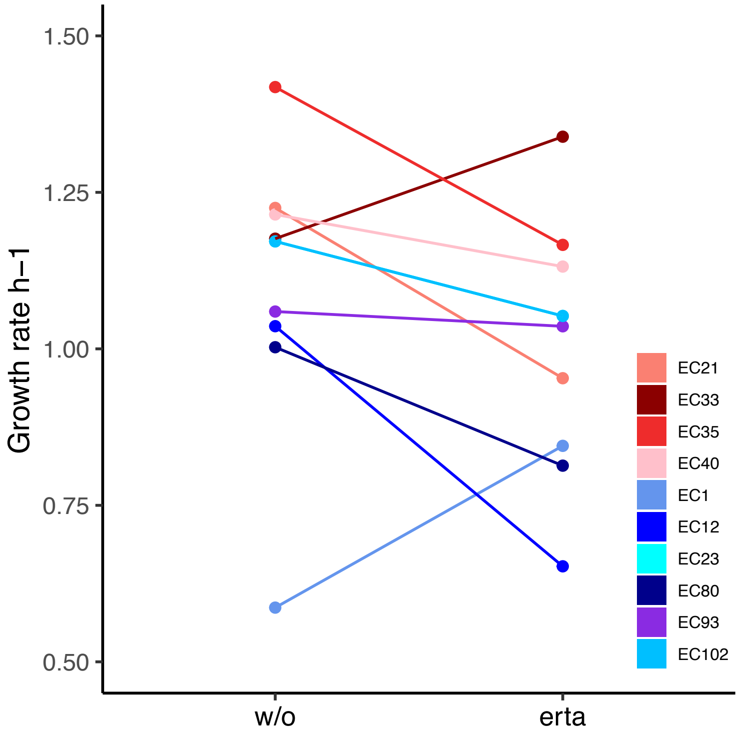

Figure S2. Maximum specific growth rates of all strains grown without (w/o) and with (erta) ertapenem. Growth rates of EC23 and EC102 were very similar and are not distinguishable on the plot.

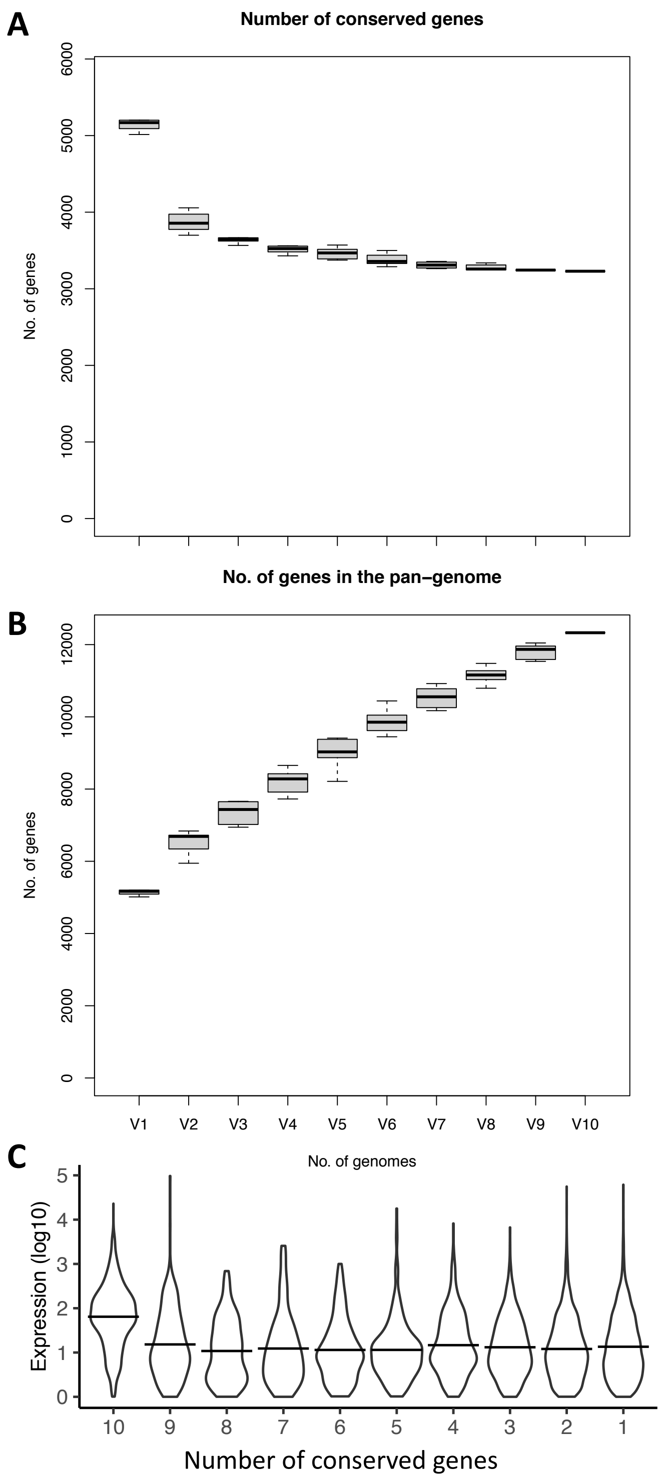

Figure S3. Pangenome analysis of strains from this study. Panel A displays the number of conserved genes, where results of genomes are added consecutively. The corresponding plot for pan genes (all unique genes) is shown in B. Panel C shows the average expression levels (TPMs) of genes of individual categories of conservation (e.g. 10=present in all strains; 1=present in only one strain).
